# Eating versus skipping breakfast has no discernible effect on obesity-related anthropometric outcomes: a systematic review and meta-analysis

**DOI:** 10.12688/f1000research.22424.2

**Published:** 2020-10-15

**Authors:** Michelle M. Bohan Brown, Jillian E. Milanes, David B. Allison, Andrew W. Brown

**Affiliations:** 1 Department of Applied Health Science, Indiana University School of Public Health - Bloomington, Bloomington, IN, 47405, USA; 2 Department of Genetics and Biochemistry, Clemson University, Clemson, SC, 29634, USA; 3 Department of Epidemiology and Biostatistics, Indiana University School of Public Health - Bloomington, Bloomington, IN, 47405, USA

**Keywords:** Breakfast, skipping, obesity, weight, meta-analysis, systematic review, randomized controlled trials

## Abstract

**Background:** Eating or skipping breakfast for weight interests scientific and lay communities. Our objective was to systematically review and meta-analyze causal effects of eating versus skipping breakfast on obesity-related anthropometric outcomes in humans.

**Methods:** Six databases were searched for obesity- and breakfast-related terms (final search: 02 JAN 2020). Studies needed to isolate eating versus skipping breakfast in randomized controlled trials. Mean differences were synthesized using inverse variance random effects meta-analysis for each outcome. Positive estimates indicate higher outcomes in breakfast conditions (e.g., weight gain). Leave-one-out analysis for sensitivity and a secondary baseline habit-by-breakfast assignment analysis were performed. Risk of bias was assessed using the Cochrane risk of bias tool.

**Results:** Ten articles (12 comparisons; 6d to 12wk) were included. Conditions included recommendations to eat versus skip breakfast, or provision of some or all meals. 95% confidence intervals of all main analyses included the null value of no difference for each outcome: body weight (0.17 kg [-0.40,0.73], k=12, n=487, I ^2^=74.5), BMI (0.07 kg/m ^2^ [-0.10,0.23, k=8, n=396, I ^2^=54.1), body fat percentage (-0.27% [-1.01,0.47], k=6, n=179, I ^2^=52.4), fat mass (0.24 kg [-0.21,0.69], k=6, n=205, I ^2^=0.0), lean mass (0.18 kg [-0.08,0.44], k=6, n=205, I ^2^=6.7), waist circumference (0.18 cm [-1.77,2.13], k=4, n=102, I ^2^=78.7), waist:hip ratio (0.00 [-0.01,0.01], k=4, n=102, I ^2^=8.0), sagittal abdominal diameter (0.19 cm [-2.35,2.73], k=2, n=56, I ^2^=0.0), and fat mass index (0.00 kg/m ^2 ^[-0.22,0.23], k=2, n=56, I ^2^=0.0). Subgroup analysis showed only one statistically significant result. The interaction effect for BMI (–0.36[-0.65,-0.07]) indicates assignment to conditions consistent with baseline habits had lower BMI. Leave-one-out analysis did not indicate substantial influence of any one study.

**Conclusions:** There was no discernible effect of eating or skipping breakfast on obesity-related anthropometric measures when pooling studies with substantial design heterogeneity and sometimes statistical heterogeneity.

**Registration:** PROSPERO CRD42016033290.

## Introduction

Whether one should eat or skip breakfast for weight control or loss is a topic of continued interest in both the scientific and lay communities. In 2013 ^1^, we documented how breakfast eating versus breakfast skipping served as an example of how beliefs about diet can go beyond the evidence within and beyond the scientific community. The evidence at the time was dominated by over 90 observational studies – most cross-sectional – leading us to conclude that eating versus skipping breakfast as a strategy for weight was a presumption: a belief “held to be true for which convincing evidence does not yet confirm or disprove their truth” ^2, 3^. The limited scientific evidence on the topic has been translated directly to the public. For instance, we noted in our prior paper that the website of the Dr. Oz Show included an article stating, “The fact is, when you’re trying to lose body fat, you can’t skip breakfast” ^4^. More recently, Dr. Oz himself stated, "I think for 2020, the first thing I’m going to do is ban breakfast” ^5^, and using the social media hashtag of #TeamNoBreakfast. Meanwhile, continued scientific interest in the topic is evidenced by many more cross-sectional observational and other studies having been published; more recent narrative review articles summarizing existing literature on the topic ^6, 7^; a meta-analysis evaluating breakfast eating versus skipping on weight ^8^ that confirmed our prior registered preliminary analyses ^9, 10^; and another group registering an analysis similar to ours after our registration (PROSPERO CRD42018110858; subsequently published ^11^.

With mixed messaging over time about the importance of eating or skipping breakfast for the ongoing obesity epidemic, and with continued interest in the topic both scientifically and generally, it is important to synthesize the causal evidence on the effect of breakfast eating versus skipping on obesity and related outcomes, rather than relying on weaker study designs or popular opinion.

Since our earlier summaries, additional RCTs have been conducted and published (as reviewed herein). Herein, we extend our prior work to synthesize causal evidence from RCTs on eating versus skipping breakfast in humans on all reported obesity-related anthropometric outcomes we were able to extract from relevant literature.

## Methods

### Registration

Our study was registered with the PROSPERO international prospective register of systematic reviews ( CRD42016033290) on 21 JAN 2016. The initial registration limited papers up to the registration date; however, because of the time between initial registration and this manuscript, the search was updated to 02 JAN 2020 (see *Search and review strategy*, below). Earlier versions of this work were published as abstracts for the American Society for Nutrition’s Annual Meeting and Scientific Sessions ^9, 10^.

### Inclusion and exclusion criteria

Inclusion criteria were:

- the study had at least one breakfast skipping condition and one breakfast eating condition regardless of modality (e.g., whether recommended or provisioned);
- the study was a randomized, controlled trial (RCT);
- study length (i.e., time on intervention) was greater than 72 hr;
- participants were normal weight or greater, as defined by original study authors, who did not have diseases that influence weight; and
- the study reported weight or other anthropometric outcomes.

Studies were excluded if:

- participants had diseases or conditions that affected weight except for obesity, diabetes, and CVD;
- breakfast eating versus breakfast skipping were confounded with other intervention components (e.g., study designs that altered intake to ensure individual weight maintenance; multicomponent behavioral interventions including breakfast without matching those components in the skipping group; or enforcing time restricted feeding apart from breakfast skipping).

### Search and review strategy

Our first search was completed on 20 JAN 2016, the search refreshed on 26 JAN 2017, and the search finalized on 02 JAN 2020, with results from prior searches being deduplicated from subsequent searches.

In all search phases, searches were executed by using the application programming interfaces (APIs) of AltHealthWatch, CINAHL, Proquest Theses and Dissertations Global, PsycInfo, and Scopus using R (version 3.5.2). The following was used to search Scopus, with analogous search strategies adapted for the other databases:

TITLE-ABS-KEY((Obesity OR obese OR adipose OR adiposity OR overweight* OR "over weight*" OR "weight gain*" OR "weight reduc*" OR "weight los*" OR "weight maint*" OR "weight decreas*" OR "weight control*" OR "weight restrict*" OR "BMI" OR "FMI" OR "BMIz" OR "zBMI" OR "weight percentile" OR "gestational weight" OR "weight for height" OR "waist circumference" OR "skinfold thickness" OR "body composition" OR "body size" OR "fat mass" OR "body fat" OR "body mass" OR "body weight" OR "bodyweight" OR "waist hip ratio") AND (breakfast OR "break fast" OR "morning fasting" OR "morning meal")) AND DOCTYPE(ar OR ip) AND SRCTYPE(j)

Search results across databases were compared for duplication, including by title, abstract, and PubMed ID. Studies with titles and abstracts addressing animals that did not also include words related to human subjects were excluded programmatically. Titles and abstracts were then coded independently by at least two authors for inclusion/exclusion criteria. If both authors excluded a study for violation of any inclusion or exclusion criterion, it was excluded; if at least one did not exclude it, the paper was passed on for full text review.

### Meta-analysis

All data and code used to estimate effect sizes and meta-analyses are provided as *Extended data* at https://doi.org/10.5281/zenodo.4041392 ^12^. Additional details are included as comments within the code, including exact approaches to estimating each effect size within a study.

Effect sizes comparing breakfast eating versus skipping on each outcome were calculated for each study. Each effect size was calculated as a difference-in-difference in the native units of the outcome (e.g., kg for weight). Only outcomes for which there was more than one effect size were meta-analyzed: body weight, BMI, body fat percentage, fat mass, lean mass, fat free mass, adipose tissue mass, waist circumference, waist:hip ratio, fat mass index, sagittal abdominal diameter, and lean tissue mass. Lean mass, fat-free mass, and lean tissue mass were meta-analyzed together as ‘lean mass’; fat mass and adipose tissue mass were meta-analyzed together as ‘fat mass’. Total body water percentage and muscle mass are both reported only in Ogata *et al.* ^13^; although muscle mass as an outcome was excluded, Ogata *et al.* also reported lean mass, which is captured in the pooled lean mass analysis.

Farshchi *et al.* ^14^ reported pre and post means and standard deviations separately for each treatment period in a two-arm cross-over design. Although the unbiased estimate of the difference-in-difference was calculable from the pre and post means in each condition, the lack of information on the correlation of change within or between conditions precluded us from directly calculating the variance of the effect. We requested summaries from the authors, but the authors informed us they no longer had the raw data given that the paper was published in 2005. Thus, within-condition and between-condition correlations had to be estimated. Sievert *et al.* ^8^ used a correlation coefficient of 0.3 for post-only values. We chose to estimate within-period change scores based on the within-condition correlation coefficients we estimated from Geliebter *et al.* ^15^ because Geliebter *et al.* had all values needed to estimate within-condition, pre-post correlation coefficients. All correlation coefficients from Geliebter were greater than 0.99. Effect sizes were estimated for each outcome. Because Farshchi *et al.* reported no statistically significant results for any outcome, any statistically significant estimates were recalculated using the largest within-condition correlation that resulted in non-significant effect sizes. This approach may underestimate the variance, which would provide the study more weight in the meta-analysis; however, the leave-one-out analysis described below gives Farshchi the lowest weight possible.

Geliebter *et al.* ^15^ reported three conditions: skipping, corn flakes, and oat porridge. We used the recommended method of the *Cochrane Handbook*, which is to “combine multiple groups that are eligible as the experimental or comparator intervention to create a single pair-wise comparison” ^16^. Because we were interested in breakfast eating versus breakfast skipping, the two breakfast conditions were pooled together.

Leidy *et al.* ^17^ also reported three conditions: skipping, a normal protein breakfast, and a high protein breakfast. We requested summaries from Leidy *et al.*, who graciously provided us with separate group means and standard deviations for the changes. We used the recommended method of the *Cochrane Handbook* to combine breakfast conditions as described above.

Neumann *et al.* ^18^ reported three conditions: skipping, high carbohydrate breakfast, and high protein breakfast. Again, we used the method recommended by the *Cochrane Handbook* to combine breakfast conditions. Neumann *et al.* reported individual-level data in their supplementary table. While reviewing the values in their supplement, we found some results to be implausible. We reached out to the authors, who clarified several subjects’ data. For our analysis, we used the updated values, and include the final updated dataset in our supplement.

Schlundt *et al.* ^19^ reported follow-up data at 6 months, but the methods descriptions were unclear as to whether the interventions to eat or skip breakfast were continued past the 12-week intervention. Authors were contacted about this detail and for additional outcomes data at 12 weeks that were either not directly reported or reported as no significant strata (i.e., habitual breakfast eaters or skippers) or treatment effects; the authors informed us they no longer had the raw data given the study was published in 1992. We therefore chose to only use the change in body weight data from 12-weeks. Independent effect sizes were estimated for habitual breakfast eaters and habitual breakfast skippers.

Dhurandhar *et al.* ^20^ reported body weight for the completers-only analysis in their paper. Because they registered their study as also measuring BMI, and because of the mention of an intention to treat analysis, we contacted the authors (one of whom, DBA, is a coauthor on the present meta-analysis), who provided us with summary data. Note that they also had a third group, in which participants received no specific breakfast eating or breakfast skipping recommendations; we limited our analysis to the intention to treat analyses of the breakfast eating and breakfast skipping groups. Independent effect sizes were estimated for habitual breakfast eaters and habitual breakfast skippers.

LeCheminant *et al.* ^21^ were contacted for estimates of change over time for data in their Table 3. The authors graciously provided estimates of change within each group for each outcome. The data used herein, as shared by the authors, differs slightly from their publication because of increased precision and because of a reporting error in which percent body fat did, in fact, have a small but non-significant increase in the no breakfast group. This error does not change the results of their study, but the corrected values are used herein.

Ogata *et al.* ^13^, Betts *et al.* ^22^, and Chowdhury *et al.* ^23^ effect sizes were calculated with routine equations.

Meta-analyses were calculated using the metafor package(version 2.1-0) in R. Each of 12 independent effects sizes (10 papers; 2 stratified by baseline habit) were included in each analysis as possible, depending on which outcomes were reported in which studies. Random effects analyses were calculated; no fixed effects analyses were calculated because design heterogeneity made the assumption of effect sizes being part of a homogenous distribution tenuous. The adjustment by Knapp and Hartung ^24^ was used given the relatively small number of effect sizes. Two effect sizes were derived from separate papers of the Bath Breakfast Project (BBP; Betts *et al.* and Chowdhury *et al.*). Because these were independent samples (normal or with obesity) we treated them as independent even though they came from the same overarching study. Similarly, although there is plausibly some correlation amongst effect sizes calculated within the habit strata in Dhurandhar *et al.* and Schlundt *et al.* by nature of being part of the same overarching study, we treated the effect sizes as independent.

Leave-one-out analysis was used as a sensitivity analysis to investigate the influence of any single study for each outcome, in which each study was omitted from the dataset at a time, and then the meta-analysis was recalculated.

Effect estimates are displayed as mean differences with 95% confidence intervals in the native units of the outcome. I ^2^ (%) and p-values for tests of heterogeneity are also reported. No multiple-comparison corrections are applied within or among outcomes. There are few effect sizes (k=12), there is substantial design heterogeneity (e.g., study length, types of breakfast, different populations), and there is statistical heterogeneity in several outcomes; therefore, funnel plot asymmetry is not presented because visual estimation of asymmetry is unreliable for small k ^25^, the test is underpowered for small k ^26^, and any association between effect size and variance may plausibly be explained by study design or other factors rather than just publication bias ^26^.

We calculated a secondary analysis comparing habitual breakfast eaters versus habitual breakfast skippers, as defined by the authors of the published papers. This subgroup analysis completed the preliminary analyses we initially published as abstracts ^9, 10^. If baseline habits were not reported, or if authors reported that the baseline habits of the participants were mixed, the study arms were excluded. The subgroup analysis was based on the same effect size estimates generated for the primary analysis. The same functions were used in R, except for modeling the outcome variable as a function of both assigned condition and baseline breakfast habits. Additional methods can be found in the subgroup analysis document and code located in https://doi.org/10.5281/zenodo.4041392.

### Risk of bias

Risk of bias was assessed independently by two investigators (MMBB/JEM for all but Ogata 2019 and MMBB/AWB for Ogata 2019) using Cochrane’s Risk of Bias Tool ^27^. Given that the interventions are obvious to participants (eating versus skipping breakfast), we only coded blinding of personnel, and readers should be aware of the risk of non-blinded interventions. We do not use the approach of assigning a binary risk of bias to an entire study (e.g., if one criterion is high risk in a study, the entire study is considered high risk); however, we provide the individual ratings for each article and readers can apply such an approach if they wish.

## Results

### PRISMA diagram

**Figure 1.**
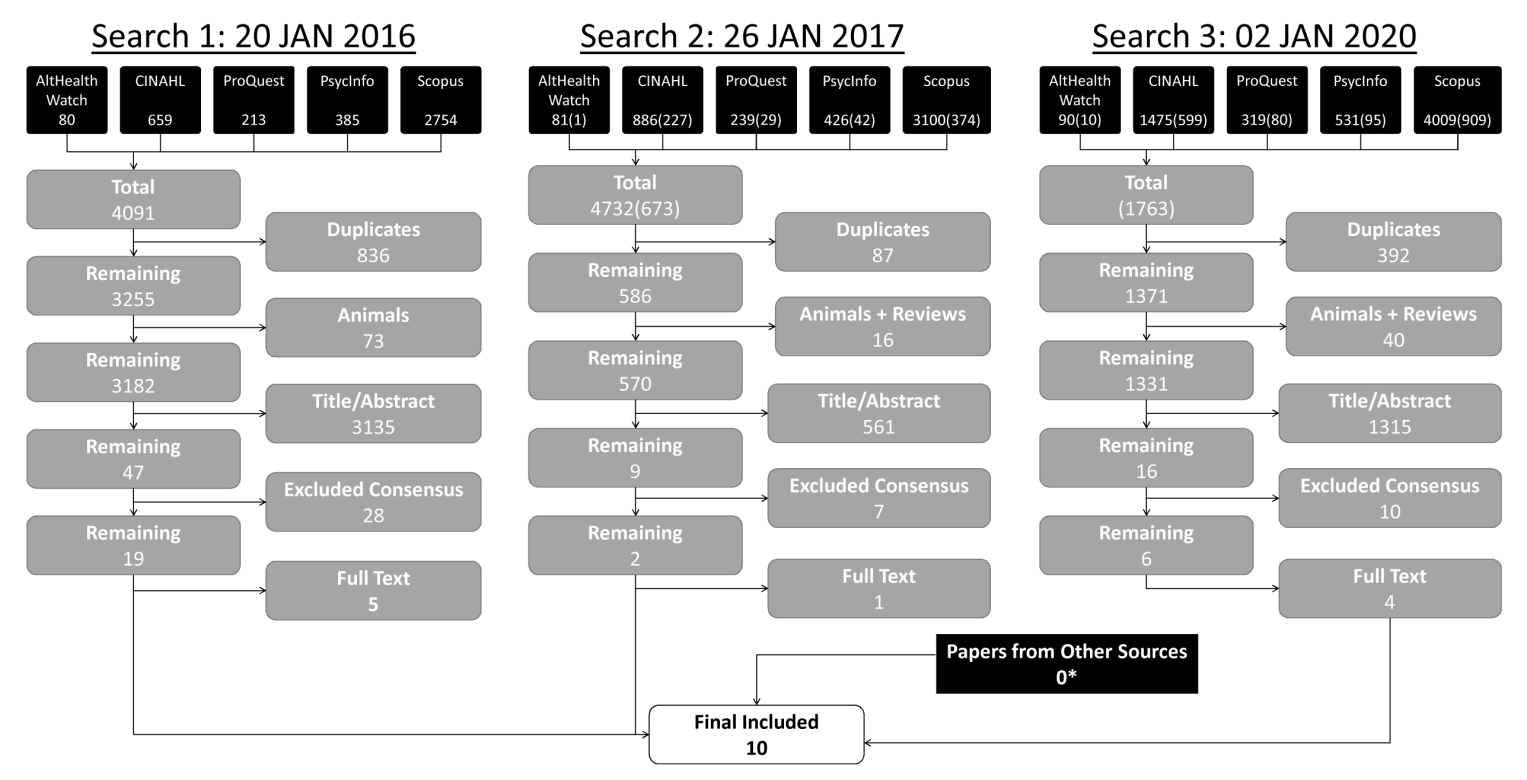
PRISMA diagram. Three searches were undertaken. For searches 2 and 3, the numbers in parentheses represent unique results to that search. *Several ‘papers from other sources’ were identified in prior searches, but those papers were captured by the third search.

The search results are shown in the PRISMA diagram in Figure 1. The results of each of the three phases of the search are shown.

### Inclusion table

**Figure 2.**
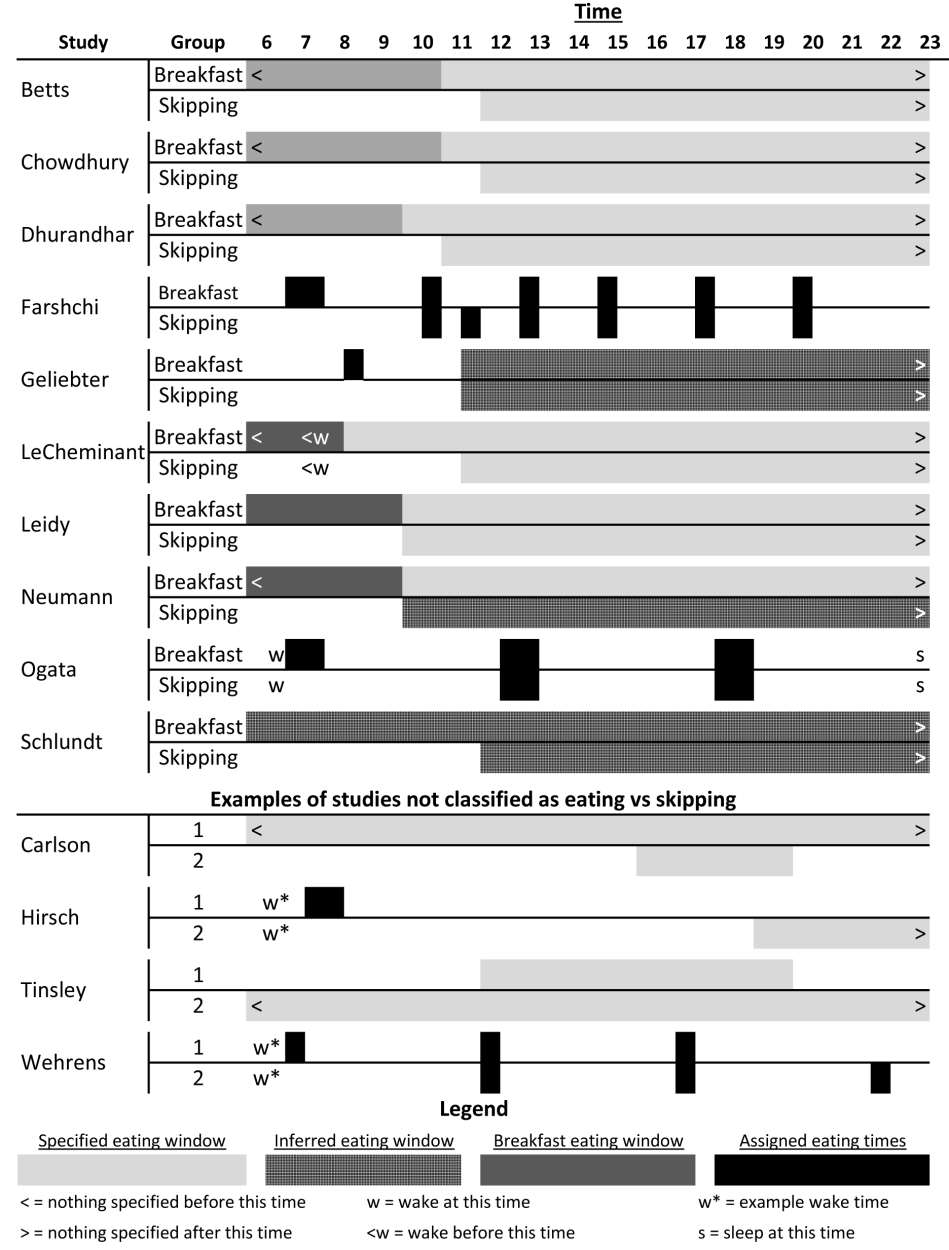
Schematic of breakfast versus skipping timing and patterns. The top section outlines the patterns for the included studies; the middle section shows a few examples of studies we did not classify as eating versus skipping breakfast that are explained further in the ‘Notable Exclusions’ section and in Table 3; and the bottom is a legend for the figure. ‘Inferred eating window’ represents the times we inferred that participants were permitted or recommended to consume food as reported in the papers; ‘specified eating window’, ‘breakfast eating window’, and ‘assigned eating times’ were reported by the authors in either absolute or relative times (e.g., number of hours since waking). For more details for the included studies, see Table 1.

Ten papers were included with 12 effect sizes (see Table 1 for descriptions). Briefly, of the 10 studies included: six were conducted in the United States, three in the United Kingdom, and one in Japan; two were cross-over RCTs and eight were parallel arm RCTs; length ranged from 6 days to 16 weeks; five provisioned some or all foods and five were recommendations for dietary consumption; two stratified on baseline eating or skipping habits, two included only habitual breakfast eaters, three included only habitual breakfast skippers, two reported mixed baseline habits, and one did not specify baseline habits; four reported race/ethnicity of participants; four included females only, one included males only, and five included both females and males. For breakfast definitions, dietary compositions, and timing, see Table 1 and Figure 2. Breakfast definitions and timing of consumption varied amongst the studies included and ranged from highly controlled and prescribed to broad recommendations ( Figure 2).

**Table 1.** Included studies.

Study	Location	Population	Age (Mean ± SD) ^1^	Race/Ethnicity ^2^	Intervention	Provision of Food	Baseline Breakfast Habits (Eaters vs Skippers)	Breakfast Eating and Breakfast Skipping Definitions ^3^	Dietary Composition ^3^	Weight-related anthropometric measures preregistered as primary or secondary outcome	Weight-related anthropometric measures reported ^4^
Betts 2014	UK	Adults: n=33 64% Female 21 – 60 y	All: 36 ± 11 y BF: 36 ± 11 y Skip: 36 ± 11 y	Not reported	6 wk parallel arm RCT Recommendation to eat or skip breakfast	No	Mixed	Breakfast group: consume energy intake of ≥700 kcal before 1100h daily, with at least half consumed within 2 h of waking Fasting group (skip): Extend overnight fast by abstaining from ingestion of energy- providing nutrients (plain water only) until 1200 h each day.	No recommendation for the diet was given.	Yes: ISRCTN31521726	BW, BF%, BMI, ATM, FMI, LTM, SAD, WC, WHR
Chowdhury 2016	UK	Adults: n=23 65% Female 21 – 60 y	All: 44 ± 10 y BF: 44 ± 10 y Skip: 44 ± 10 y	Not reported	6 wk parallel arm RCT Recommendation to eat or skip breakfast	No	Mixed	Breakfast group: consume energy intake of ≥700 kcal before 1100h daily, with at least half consumed within 2 h of waking Fasting group (skip): Extend overnight fast by abstaining from ingestion of energy- providing nutrients (plain water only) until 1200 h each day.	No recommendation for the diet was given.	Yes: ISRCTN31521726	BW, BF%, BMI, ATM, FMI, LTM, SAD, WC, WHR
Dhurandhar 2014	USA	Adults: n=185 76% Female 20 – 65 y	BF: 40.6 ± 12.0 y Skip: 42.0 ± 12.4 y	Total: WHN: 93, BNH:74, WH:17, BH:8, O:12 Breakfast: WHN: 45, BNH:40, WH:5, BH:5, O:6 Skip: WHN: 48, BNH:34, WH:12, BH:3, O:6	16 wk parallel arm RCT Recommendation to eat breakfast, skip breakfast, or neither (control group); all three treatment groups were given a USDA pamphlet suggesting good nutrition habits in baseline skippers and eaters	No	Stratified	Breakfast Eating: meal before 1000h. Skipping: no eating or caloric consumption prior to 1100 h.	The breakfast group received the USDA pamphlet with a handout instructing participants to consume breakfast before 1000 h every day. The breakfast handout also provided suggestions of food items that might constitute a healthy breakfast; however, no specific restrictions were given on types of foods that could be consumed for the breakfast meal. The skipping group received the USDA pamphlet with a handout instructing participants not to consume any calories before 1100 h every day, and that only water or zero-calorie beverages could be consumed from the time of waking until 1100 h. No specific composition was recommended.	Yes: NCT01781780	BW, BMI
Farshchi 2005	UK	Adults: n=10 100% Female 19 – 38 y	Total: 25.5 ± 5.7 y	Not reported	2 wk per condition, cross- over RCT Intervention program to eat or skip breakfast	Breakfast and one snack	Habitual eaters	Breakfast between 0700h and 0800h. Skipping nothing prior to 1030 h.	Breakfast group consumed a pack (45 g) of whole-grain cereal with 200 mL 2% milk between 0700 h and 0800 h. and consumed a chocolate- covered cookie between 1030 h and 1100 h. Skippers had nothing prior to both groups consuming a 48-g chocolate- covered cookie between 1030 h and 1100 h. Skippers then had the cereal and 2%-fat milk between 1200 h and 1230 h. Both groups then consumed 2 additional meals and 2 snacks of content similar to usual during the times of 1330–1400, 1530–1600, 1800–1830, and 2030–2100. Subjects were asked to consume their main evening meal (dinner) between 1800 and 1830.	Not registered	BW, BF%, BMI, WC, WHR
Geliebter 2014	USA	Adults: n=36 50% Female 8 – 65 y	Total sample: 33.9 ± 7.5 y M:35.6 ± 6.1 y F: 32.3 ± 8.6 y	Total: W:16, B:10, H:6, A:3, O:3 Skip: W:4, B:3, H:3, A:1, O:1 C: W:6, Breakfast:3, H:2, A:2, O:1 P: W:6, B:4, H:1, A:0, O:1	4 wk parallel arm RCT Recommendation to skip breakfast compared to provision of high fiber (oat porridge) and non-fiber (cornflakes) breakfasts	Breakfast only	Unspecified	0830 h arrival weekdays with 15 min given to consume breakfast or water for skip group. Breakfasts were given to take home for weekends with no time given on weekends	No recommendation for the remainder of the diet was given.	Registered after: NCT02035150	BW, FFM, FM, WC, WHR
LeCheminant 2017	USA	Adults: n=49 100% Female 18 – 55 y	BF: 23.7 ± 7.5 y Skip: 23.6 ± 5.0 y	Not reported	4 wk parallel arm RCT Recommendation to eat or skip breakfast in habitual skippers	No	Habitual skippers	Breakfast group to eat within 1.5 h of awakening and consume 15% total energy intake for the day by 0830 h. Skippers were defined as not consuming a snack or meal (only noncaloric beverages) until after 1130 h.	No recommendation for the remainder of the diet was given. Both groups asked to wake up by 0800.	Not registered	BW, FM, LM, BF%, BMI
Leidy 2015	USA	Adolescent: n=54 57% Female 19 y (mean)	Skip:19 ± 1 y Normal Protein BF: 18 ± 1 y High Protein BF: 19 ± 1 y	Total: W:33, B:19, O:2 Skip: W:6, B:3, O:0 Normal Protein: W:16, B:5, O:0 High Protein: W:11, B:11, O:2	12 wk parallel arm RCT Recommendation to skip breakfast compared to the provision of normal protein and high protein breakfasts in habitual skippers	Breakfast only	Habitual Skippers	Breakfast consuming groups were provided with specific breakfast meals with consumption of breakfast between 0600 h and 0945 h each day. The skipping group continued to skip breakfast (only water) before 1000.	The NP meals contained 15% protein, 65% carbohydrates, and 20% fat and consisted of ready-to-eat cereals with milk. The HP meals contained 40% protein, 40% carbohydrates, and 20% fat and consisted of egg-based pancakes and ham; egg-based waffles with pork-sausage; egg and pork scramble; and an egg and pork burrito. The breakfast meals were provided on a weekly basis with meal preparation instructions. Breakfasts were 18% of total dietary calories. No recommendation for the remainder of the diet was given.	Not registered	BW, FM, LM, BF%, BMI
Neumann 2016	USA	Adults: n =24 100% Female 11 – 36 y	Skip: 27.1 ± 1.8 y Carbohydrate BF: 21.9 ± 0.9 y Protein BF: 23.3 ± 1.3 y	Skip: C:5, H:1, B:1, A:0, I:1 Carbohydrate: C:3, H:1, B:1, A:2, I:1 Protein: C:6, H:1, B:1, A:0, I:0	8 d parallel arm RCT Assignment to skip or eat breakfast with provision (breakfast or water) in habitual skippers	Breakfast only	Habitual skippers	Breakfast group: eat breakfast before or at the start of daily activities and within two hours of waking with consumption typically occurring no later than 1000 h. Skipping group: provided water with no other instructions given.	Breakfast: CHO breakfast consisted of 1 English muffin (57 g), yogurt (170 g), cream cheese (17g), and water (227 mL). The PRO breakfast consisted of a proprietary breakfast sandwich (145 g), Greek yogurt (150g), and water (227 mL). Both test breakfasts were similar in kilocalories and controlled for fat and fiber. Skipping group was provided water (227 mL). No recommendation for the remainder of the diet was given.	Not registered	BW, BMI
Ogata 2019	Japan	Adult: n=10 0% Female 20 – 30 y	BF to Skip: 24.8 ± 2.9 y Skip to BF: 25.6 ± 3.0 y	Japanese:10	6 d per condition, cross-over RCT Intervention to eat or skip breakfast	All food	Habitual eaters	Breakfast eating group consumed breakfast at 0700 h, breakfast skipping group nothing prior to lunch at 1230 h.	Breakfast eating group had 33.3% of daily energy intake for each of the three meals of breakfast (0700 h), lunch (1230 h) and dinner (1800 h). The breakfast skipping group had 0% for breakfast, 50% of daily energy intake each for lunch (1230 h) and dinner (1800h). The 24-h energy intake was equal for both dietary conditions. The meals provided were individually adjusted (3042 ± 598 kcal/d, 14% protein, 25% fat, and 61% carbohydrates).	Yes: UMIN000032346	BW, BF%, FM, FFM, MM, TBWP
Schlundt 1992	United States	Adults: n= 45 100% Female 18 – 55 y	Only range stated	Not reported	12 wk parallel arm RCT Baseline breakfast eaters and skippers were assigned to either eat or skip breakfast with total diet composition and caloric content same between groups	No	Stratified	Menus and instructions for 3 meals (breakfast, lunch and dinner) or 2 meals (lunch and dinner), timing not specified in the paper.	Total dietary composition: 50–55% of energy from carbohydrates, 15–20% from protein, and 25–30% from fats. No- breakfast diet consisted of two meals, lunch (1672 kJ) and supper (3344 kJ). Breakfast diet consisted of three meals, breakfast (1672 kJ), lunch (1254 kJ), and supper (2090 kJ).	Not registered	BW

^1^BF, Breakfast. ^2^A, Asian; B, Black; BH, Black Hispanic; BNH, Black Non-Hispanic; C: Caucasian; H, Hispanic; I, Indian; O, Other; W, White; WH, White Hispanic; WNH, White Non-Hispanic. ^3^Definitions paraphrased from each study paper. ^4^ATM, adipose tissue mass; BF%, body fat percentage; BW, body weight; FFM, fat-free mass; FM, fat mass; FMI, fat mass index; LM, lean mass; LTM, lean tissue mass; MM, muscle mass; SAD, sagittal abdominal diameters; TBWP, total body water percentage; WC, waist circumference; WHR, waist:hip ratio. Some additional outcomes might have been mentioned in the paper, but quantitative results may not have been reported after the intervention.

### Meta-analyses of anthropometric outcomes

**Figure 3.**
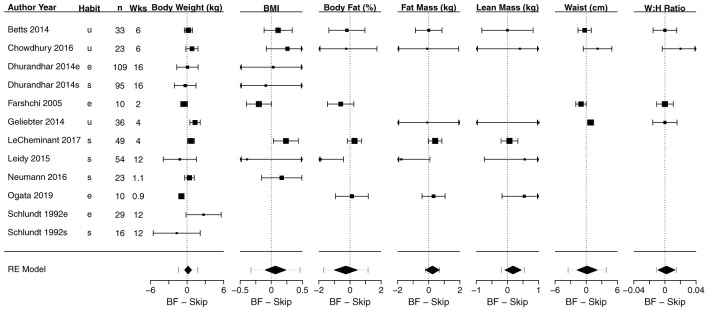
Composite forest plot of seven meta-analyzable anthropometric outcomes. Sagittal abdominal diameter and fat mass index were only included in the two papers from the Bath Breakfast Project (Betts *et al.* and Chowdhury *et al.*), and are not plotted here; outcomes of muscle mass and total body water percent were only included in Ogata *et al.*, and so no meta-analyzable estimate was possible. See Table 2 for the numerical values of these seven analyses, plus the sagittal abdominal diameter and fat mass index. Studies without point estimates and confidence intervals within an outcome indicates that the study did not report that outcome. 95% confidence intervals for individual studies and for the width of the diamond representing the summary estimate are presented. Horizontal dotted lines for the summary of the meta-analyses represents the 95% prediction interval. For the column ‘Habit’: e, habitual eaters; s, habitual skippers; u, unspecified or mixed. Values to the left of 0 indicate that the breakfast condition had a greater decrease in the outcome relative to the skipping condition. For all outcomes except lean mass, values to the left would therefore traditionally be considered 'favoring' the breakfast condition (e.g., lower body weight)

Figure 3 shows a composite forest plot that includes all meta-analyzable, obesity-related, anthropometric outcomes. In all cases, the 95% confidence intervals included the null of no differences between skipping and eating breakfast (frequently interpreted as “not statistically significant”). Table 2 shows the numerical estimates of the values displayed in the forest plots. Therefore, no discernible effects of breakfast eating or breakfast skipping on body weight (kg), BMI (kg/m ^2^), body fat percentage (%), fat mass (kg), lean mass (kg), waist (cm), waist:hip ratio, sagittal abdominal diameter (cm) and fat mass index (kg/m ^2^) were found in these primary analyses.

In the secondary analysis comparing habitual breakfast eaters versus habitual breakfast skippers (as defined by the authors of the published papers), forest plots and a summary table can be found in the subgroup between group forest plots document and subgroup between group summary table located in https://doi.org/10.5281/zenodo.4041392. Briefly, there was no discernible effect of stratification of baseline habits on four of the outcomes: body weight (4 effect sizes for habitual breakfast eaters; 4 effect sizes for habitual breakfast skippers), body fat percentage (2 and 2 effect sizes, respectively), fat mass (1 and 2 effect sizes, respectively), or lean mass (1 and 2 effect sizes, respectively). Insufficient study arms existed to test differences for waist circumference, waist:hip ratio, sagittal abdominal diameter, and fat mass index. Only BMI was statistically significant (2 and 4 effect sizes, respectively). The negative estimate of –0.36[-0.65,-0.07] BMI units indicates that individuals assigned to conditions consistent with their baseline habits had lower values than those assigned opposite of their habits. That is, habitual breakfast skippers had lower BMI when skipping breakfast and habitual breakfast eaters had lower BMI when eating breakfast, compared to skippers eating and eaters skipping.

**Table 2.** Effect sizes for each study and meta-analyzable anthropometric outcome shown in Figure 3. Data are presented as mean [95% CI] for each study and the summary estimate, expressed as mean difference. Positive values are higher during breakfast conditions. n represents the total number of individuals within a study; k is the number of effect sizes in a meta-analytic estimate; MD is mean difference; I ^2^ represents heterogeneity, with the associated p-value representing the statistical test for significant heterogeneity. Outcomes of muscle mass and total body water percent were only included in Ogata *et al.*, and so no meta-analyzable estimate was possible.

Study	n	Body weight (kg)	BMI	Body fat (%)	Fat mass (kg)	Lean mass (kg)	Waist circumference (cm)	Waist:hip ratio	Sagittal abdominal diameter (cm)	Fat mass index
Betts 2014	33	0.20 [-0.46,0.86]	0.11 [-0.12,0.34]	-0.20 [-1.36,0.96]	0.00 [-0.85,0.85]	0.00 [-0.82,0.82]	-0.30 [-1.58,0.98]	0.00 [-0.02,0.02]	0.00 [-0.64,0.64]	0.01 [-0.28,0.30]
Chowdhury 2016	23	0.80 [-0.19,1.79]	0.26 [-0.08,0.60]	-0.24 [-2.21,1.73]	-0.10 [-2.12,1.92]	0.40 [-1.63,2.43]	2.20 [-0.56,4.96]	0.02 [-0.00,0.04]	0.40 [-0.28,1.08]	-0.04 [-0.76,0.68]
Dhurandhar 2014e	109	0.06 [-1.68,1.80]	0.03 [-0.59,0.65]							
Dhurandhar 2014s	95	-0.31 [-2.09,1.46]	-0.09 [-0.72,0.54]							
Farshchi 2005	10	-0.50 [-1.07,0.07]	-0.20 [-0.40,0.00]	-0.60 [-1.45,0.25]			-1.00 [-2.00,0.00]	0.00 [-0.01,0.01]		
Geliebter 2014	36	1.30 [0.46,2.14]			-0.09 [-2.38,2.19]	1.00 [-1.24,3.24]	0.85 [0.27,1.43]	0.00 [-0.02,0.02]		
LeCheminant 2017	49	0.64 [0.09,1.19]	0.24 [0.03,0.44]	0.29 [-0.17,0.75]	0.41 [-0.03,0.85]	0.06 [-0.21,0.33]				
Leidy 2015	54	-1.20 [-3.90,1.50]	-0.39 [-1.30,0.52]	-1.91 [-3.41,-0.42]	-1.77 [-3.62,0.08]	0.55 [-0.74,1.85]				
Neumann 2016	23	0.37 [-0.41,1.16]	0.17 [-0.16,0.49]							
Ogata 2019	10	-0.93 [-1.37,-0.49]		0.12 [-0.93,1.17]	0.31 [-0.43,1.05]	0.54 [-0.18,1.26]				
Schlundt 1992e	29	2.70 [-0.19,5.59]								
Schlundt 1992s	16	-1.70 [-5.55,2.15]								
MD [CI]		0.17 [-0.40,0.73]	0.07 [-0.10,0.23]	-0.27 [-1.01,0.47]	0.24 [-0.21,0.69]	0.18 [-0.08,0.44]	0.18 [-1.77,2.13]	0.00 [-0.01,0.01]	0.19 [-2.35,2.73]	0.00 [-0.22,0.23]
k (n)		12 (487)	8 (396)	6 (179)	6 (205)	6 (205)	4 (102)	4 (102)	2 (56)	2 (56)
I ^2^ (p for I ^2^)		74.5 (<0.001)	54.1 (0.036)	52.4 (0.055)	0.0 (0.311)	6.7 (0.682)	78.7 (0.002)	8.0 (0.413)	0.0 (0.376)	0.0 (0.895)

**Table 3.** Notable studies that were excluded with reasons.

Study	Reason for exclusion *	Notes
Alwatter 2015 ^32^	No weight or anthropometry	Adolescent girls
Frape 1997 ^33^	No weight or anthropometry	Adults
Gwin 2018 ^34^	No weight or anthropometry	Adults
Halsey 2012 ^35^	No weight or anthropometry	Adults
Hoertel 2014 ^36^	No weight or anthropometry	Adolescent girls
Leidy 2013 ^37^	No weight or anthropometry	Adolescent girls
Reeves 2014 ^38^	No weight or anthropometry	Adults
Reeves 2015 ^39^	No weight or anthropometry	Adults
Rosi 2018 ^40^	Less than 72 hr	Adult men; no weight
Yoshimura 2017 ^41^	Less than 72 hr	Adult women; one-day study
Zakrewski-Frue 2017 ^42^	Less than 72 hr	Adolescent girls; only baseline weight
Carlson 2007 ^43^	Not about breakfast	Adults; did not include weight outcomes; compared 1 vs 3 meals per day with weight being deliberately maintained (see Figure 2)
Hirsch 1975 ^29^	Not about breakfast	Adults; dinner only versus breakfast only (see Figure 2)
Keim 1997 ^44^	Not about breakfast	Adult Women; distribution of calories as 70% morning versus 70% evening
Tinsley 2019 ^45^	Not about breakfast	Adult women; time-restricted feeding versus not (see Figure 2)
Wehrens 2017 ^28^	Not about breakfast	Adult men; non-randomized order; all meals (not just breakfast) shifted 5 hours (see Figure 2)
Ask 2006 ^46^	No skipping condition	Children; quasi-experiment
Crepinsek 2006 ^47^	No skipping condition	Children
Douglas 2019 ^48^	No skipping condition	Adolescent girls
Jakubowicz 2012 ^49^	No skipping condition	Adults
Powell 1998 ^31^	No skipping condition	Children
Rosado 2008 ^30^	No skipping condition	Children
St Onge 2015 ^50^	No skipping condition	Children
Versteeg 2017 ^51^	No skipping condition	Adult men
Zakrewski-Frue 2018 ^52^	No skipping condition	Adolescent girls; breakfast skipping was alternate day skipping; no weight beyond baseline
Chowdhury 2019 ^53^	Data published elsewhere	BBP: weight data in Chowdhury 2016
Gonzalez 2018 ^54^	Data published elsewhere	BBP: weight data in Betts 2014 and Chowdhury 2016
Tuttle 1954 ^55^	Confounded design	Boys, men, and women; non-counterbalanced cross- over; some participants were assigned to gain or lose weight

* Studies were excluded for at least one reason; the reasons given in this column may not be the only reason for exclusion.

### Risk of bias

**Figure 4.**
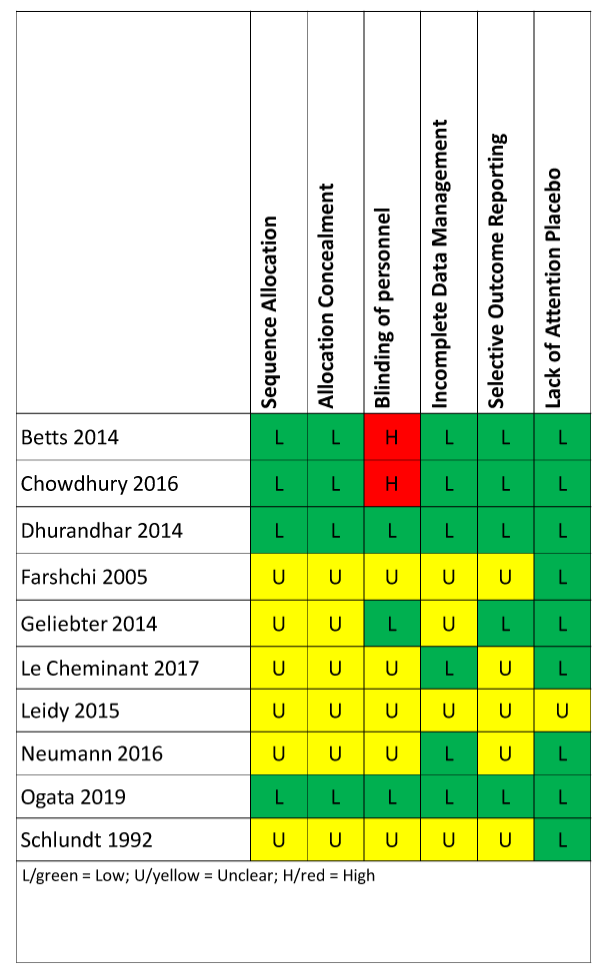
Risk of bias assessment. Each included paper was assessed for risk of bias using the Cochrane Risk of Bias tool. Given that the interventions are obvious to participants (eating versus skipping breakfast), we only coded blinding of personnel, and readers should be aware of the risk of non-blinded interventions.

Risk of bias varied by study ( Figure 4). Two studies had low risk of bias across all categories: Dhurandhar 2014 and Ogata 2019 ^13^. Two studies, Betts 2014 ^22^ and Chowdhury 2016 ^23^, were coded as high risk of bias for the criterion of blinding participants and personnel because the authors clearly indicated that personnel were not blinded. Given that the interventions are obvious to participants (eating versus skipping breakfast), we only focus on blinding of personnel, and readers should be aware of the risk of non-blinded interventions. On the other hand, many of the categories in the risk of bias in each study were unclear, and it is therefore uncertain whether the risk was high or low.

### Sensitivity analysis: Leave-one-out analysis

**Figure 5.**
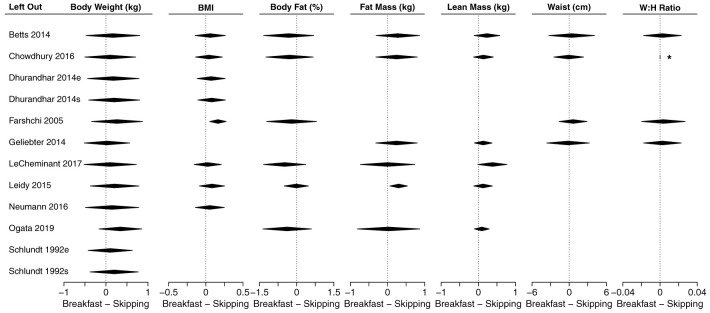
Leave-one-out analysis. Within each column, the diamond represents the meta-analytic summary estimate when leaving out the study in a particular row. Row and column combinations without diamonds represent outcomes that are not reported for that particular study. *The waist:hip ratio had no estimable confidence interval because the three remaining estimates were all 0.00. Sagittal abdominal diameter and fat mass index were only included in the two papers from the Bath Breakfast Project (Betts *et al.* and Chowdhury *et al.*), and therefore a leave-one-out analysis would include only a single study; outcomes of muscle mass and total body water percent were only included in Ogata *et al.*, and so a leave-one-out analysis is not possible.

The leave-one-out analysis is shown in Figure 5. Little difference is noted among the analyses, with substantial overlap of confidence intervals in all cases. When considering statistical significance (i.e., confidence intervals that do not include 0), leaving Farshchi *et al.* ^14^ out of the analysis results in significantly greater BMI in the breakfast conditions than the skipping conditions. When Leidy *et al.* ^17^ is excluded, fat mass is greater in the breakfast than the skipping conditions. Waist:hip ratio is centered on zero with no estimable confidence interval when Chowdhury *et al.* ^23^ is left out because the other three estimates are all 0.00. We reiterate that none of these summaries took multiple comparisons into account.

### Notable exclusions

Notable exclusions are located in Table 3. Broad areas to note are the lack of a skipping group for comparison to breakfast groups, intervention periods that were less than 72 hr in duration, studies that had the comparison of interest but did not measure body weight, and studies whose primary purpose did not isolate breakfast eating versus breakfast skipping, such as time restricted feeding and shift in consumption periods. Two examples of the latter include Wehrens *et al.*, ^28^ who shifted all meals by 5 hours (as well as not being in a randomized order), to extreme time restriction of Halberg *et al.* ^29^ who assigned only breakfast or dinner ( Figure 2).

In this meta-analysis, our included studies were all conducted in adults/adolescents, but, as noted in Table 3, there have been several related studies conducted in children; however, none of the studies in children had a true skipping group. For instance, Rosado *et al.* ^30^ had a control group with no intervention, which is not equivalent to assigning children to skip breakfast. Similarly, Powell *et al.* ^31^ did have a group that was assigned to consume a slice of orange as an attention placebo control, but again the children were not assigned to otherwise skip breakfast.

## Discussion

### Summary

The causal effect of eating versus skipping breakfast on obesity-related anthropometric outcomes was non-significantly different from zero across body weight, BMI, body fat percentage, fat mass, lean mass, waist circumference, waist:hip ratio, sagittal abdominal diameter, and fat mass index. Our results largely match our prior analyses ^9, 10^, as well as the analysis of body weight conducted by Sievert *et al.* ^8^.

The choices of inclusion/exclusion criteria, adjustments, and assumptions to use when meta-analyzing data can influence the interpretation of results, so we highlight some of our choices here. Our choice of including studies greater than 72 hours in duration (with the shortest actually included being 6 days) should avoid including experiments measuring only transient differences from hydration, glycogen, or other very acute physiological responses. Furthermore, this approach allowed us to collate all relevant experiments and enables readers to reanalyze our results with their preferred study duration cutoff should the reader so choose. We chose to define breakfast eating versus breakfast skipping according to the intentions of the original study authors where possible. This was primarily done because a common recommendation is to eat breakfast to lose weight without any other qualifications. In doing so, we included studies that 1) may or may not have been on a weight loss background, 2) included a range of weight categories, and 3) included a variety of breakfast compositions, timings, and definitions. Another limitation that was well state by one of our reviewers: “Many of the studies included in this and other SR [systematic reviews] are small, and are often conducted in participants with a mixed usual breakfast habit who are not overweight or attempting to limit their energy intake or reduce their body weight.”

While we cannot rule out that there may be some statistically significant combination of studies, subgroups, splitting-versus-pooling of different breakfasts, or different imputation strategies, we note that the results are fairly consistently centered near zero. However, Sievert *et al.*( ^8^) and Bonnet *et al.*( ^11^) both concluded small but statistically significant differences in favor of breakfast skipping. Each review included a different subset of studies, predominantly driven by duration of studies included. For one specific point of comparison, Sievert *et al.* used a different imputation strategy than we did for Farshchi *et al.* We estimated the correlations based on the estimates from Geliebter *et al.*, while constraining the interval to the statistically non-significant results. Bonnet *et al.* did not include Farshchi *et al.* because it was too short (2 weeks) for their cutoff of 4 weeks.

Our leave-one-out analyses, produced only two values that became statistically significantly different in favor of skipping breakfast: BMI when Farshchi *et al.* was excluded. The subgroup analysis of baseline breakfast habits included few effect sizes to estimate interaction effects between baseline breakfast habits and breakfast assignment. We could test for differences in 5 of the outcomes. Of these, BMI showed a significant interaction between baseline habits and assigned condition. The statistical significance of these secondary estimates should be considered within the following contexts. In the leave-one-out analyses, the 95% confidence intervals did not differ substantially from other leave-one-out analyses. In the subgroup analyses, few effect sizes were available for comparison. There were 4 treatment effect estimates each for habitual breakfast eaters and habitual breakfast skippers for body weight, while there were only 2 and 4, respectively, for BMI. Some outcomes had only 1 and 2 effect estimates for eaters and skippers, respectively. In all cases, we did not adjust for multiple comparisons because of their exploratory nature. Given the small departure of the confidence intervals from 0, it is likely these would no longer be statistically significant if multiple comparisons were considered. Even if effects turned out to be non-zero, the 95% confidence and prediction intervals of the outcomes include effect sizes of low clinical significance, and thus further work would be needed to determine if non-zero effects are actually clinically meaningful.

Despite this relative consistency in summary effect sizes, we note that there was substantial design heterogeneity. The length of studies, for instance, varied substantially. To be confident in effects of obesity-related interventions, longer term studies are desired. However, the need for longer-term studies is often to guard against concluding that early effects (weeks to months) will result in sustained weight loss over months to years. Given the overall null findings herein, suggesting a need for longer studies would serve to test whether these relatively acute null findings reflect long-term adaptations to establishing breakfast habits. In addition, some have argued that it is not merely eating versus skipping breakfast that is important, but rather that the *type* of breakfast matters (c.f., Leidy *et al.* 2016 ^7^). Such an argument does not invalidate the question asked or the findings of this meta-analysis, however. If, for instance, a breakfast of a particular characteristic is what influences weight – be it fiber content, protein, energetic load, timing from waking, or others – then the question would not be whether eating versus skipping breakfast matters; rather, research would need to test the effects of that particular breakfast versus comparator groups, whether those comparator groups be different breakfasts or no breakfast at all.

We clarify that our results are limited to obesity-related anthropometric outcomes. As stated previously, “Just because breakfast consumption may not have a statistically significant effect on weight does not make breakfast a bad recommendation” ^56^, nor does it necessarily make it a good recommendation. Our results do not inform whether eating versus skipping breakfast is of value for blood glucose control (c.f., ^57^, cardiometabolic risk factors (c.f., ^11, 15^), school performance (c.f., ^58^, or other outcomes; nor do our results inform the effects of eating versus skipping breakfast as part of a multicomponent behavioral/intensive lifestyle intervention or time restriction paradigm (e.g., early vs late time-restricted feeding). The inference of the results of this study are limited to the broad and simplistic recommendation to eat or skip breakfast to affect anthropometric outcomes.

## Conclusion

There was no discernible effect of eating or skipping breakfast on obesity-related anthropometric measures when pooling studies with substantial design heterogeneity and sometimes statistical heterogeneity in our primary analyses.

## Data availability

The data referenced by this article are under copyright with the following copyright statement: Copyright: ï¿½ 2020 Bohan Brown MM et al.

Data associated with the article are available under the terms of the Creative Commons Zero "No rights reserved" data waiver (CC0 1.0 Public domain dedication). 

### Underlying data

All data underlying the results are available as part of the article and no additional source data are required.

### Extended data

Zenodo: Supplemental files for "Eating versus skipping breakfast has no discernible effect on obesity-related anthropometric outcomes: a systematic review and meta-analysis.". http://doi.org/10.5281/zenodo.4041392 ^12^.

This project contains the following extended data:

- calculations.R (calculates individual effect sizes for each study)
- metaanalysis with subgroup.R (reproduces the composite forest plot, leave-one-out plot, the summary table, and the subgroup analyses)
- neumann2016.csv (contains the raw data from Neumann 2016 with authors’ corrections)
- rho estimates for farshchi.R (uses data from Geliebter et al. to estimate within-condition pre-post correlations)
- Subgroup analysis - methods and results.pdf (provides the methods, results, summary table, and forest plots for the subgroup interaction estimates between baseline habits and assigned breakfast conditions)

### Reporting guidelines

Zenodo: PRISMA checklist for "Eating versus skipping breakfast has no discernible effect on obesity-related anthropometric outcomes: a systematic review and meta-analysis". http://doi.org/10.5281/zenodo.4041392 ^12^.

Data are available under the terms of the Creative Commons Attribution 4.0 International license (CC-BY 4.0).
