# Supplementary material for: Eating versus skipping breakfast has no discernible effect on obesity-related anthropometric outcomes: a systematic review and meta-analysis
Source: F1000Res. 2021 Jul 30;9:140. Originally published 2020 Feb 24. [Version 3] doi: 10.12688/f1000research.22424.3 (PMC8924556; doi:10.12688/f1000research.22424.3)
Supplement: Peer review history

# Peer review: Reviewer response for version 3

**DOI:** 10.5256/f1000research.57931.r122860

**Published:** 2022-03-15

**Authors:** Bruce Bailey

**Affiliations:** 1 Department of Exercise Sciences, Brigham Young University, Provo, UT, USA

I am reviewing the manuscript “Eating versus skipping breakfast has no discernible effect on obesity-related anthropometric outcomes: a systematic review and meta-analysis.” The paper reviews the randomized studies that examine the impact breakfast skipping vs. breakfast eating on weight and body composition related outcomes. This was a well written paper and addresses a topic that is interesting. I am not an expert on meta-analyses and am not able to comment on the best practices. The techniques did seem reasonable and I did not see any limitations but the paper should probably be evaluated by someone who is better versed in this type of research technique.

 I am generally supportive of the publication with a few comments and questions.

 Introduction

1. I thought the introduction was well written and set the table well for the paper.
2. In the first paragraph you referenced you prior registered preliminary analysis. Was this published? There is no previous mention of this analysis and it is a little confusing why you are mentioning it. Can you give more context?
3. At the end of the first paragraph I think you need a closed bracket.

Methods

1. The methods are well done and described the study well. I did not see any major omissions. I like the commentary on each study that was included in the analysis. I think this helped to describe which studies needed additional info and if the authors responded to author contact.
2. It should be noted that I am not trained in meta-analytical methods and have not participated in this type of research for 15 years. Thus it is hard for me to be critical of the research methods applied in this study. However, the methods do look reasonable and I have no concerns about them.

Results

1. The results were thoroughly explained. I appreciated the various analyses and how the authors examined the data.
2. Was data evaluated for any gender differences?
3. Did the BMI of participants in the various studies alter the outcome? Is this something that can be evaluated with the current research? Do obese respond differently than normal weight participants?
4. The figures and tables were clear and well described.

Discussion

1. The discussion we well written and I have no major concerns.
2. Could you include some comments on where you think research in this area is lacking and what needs to be done in the future??

Is the work clearly and accurately presented and does it cite the current literature?

Yes

If applicable, is the statistical analysis and its interpretation appropriate?

I cannot comment. A qualified statistician is required.

Are all the source data underlying the results available to ensure full reproducibility?

Yes

Is the study design appropriate and is the work technically sound?

Yes

Are the conclusions drawn adequately supported by the results?

Yes

Are sufficient details of methods and analysis provided to allow replication by others?

Yes

Reviewer Expertise:

Lifestyle medicine, disease prevention, weight management

I confirm that I have read this submission and believe that I have an appropriate level of expertise to confirm that it is of an acceptable scientific standard.

# Peer review: Reviewer response for version 2

**DOI:** 10.5256/f1000research.29951.r73123

**Published:** 2020-12-03

**Authors:** Nerys Astbury

**Affiliations:** 1 Nuffield Department of Primary Care Health Sciences, NIHR Oxford Biomedical Research Centre, University of Oxford, Oxford, UK

Although some changes have been made to this manuscript, I do not believe that some of the authors arguments are valid.

 If the purpose of the review was to review the literature to determine whether breakfast has an effect on obesity related anthropometric measures -  studies of 72 hours are not useful as they argue. If this were true, weight loss diets would be in this time range - whereas it is generally accepted that in order to see any clinically meaningful weight loss 12+ of changes in behaviour is required, although I do admit changes may be apparent before this.

 The other point that the authors have not adequately addressed is related to the differentiation between studies that simply recruit (usually normal weight) individuals who are not trying to lose weight or are not advised an energy restricted diet and studies nested within an active weight loss attempt.

 This is so important because eating/skipping breakfast may not have any impact in those who are not trying to restrict energy. However, in those who are trying to actively lose weight eating/skipping breakfast may have effects on appetite and regulation of energy intake may influence adherence to such strategies, thus having impact on weight change and anthropometric measures. This should be carefully discussed because many of the RCT of breakfast are the former (weight stable, healthy weight individuals), and looking at an effect of bw and body fatness in this population is to me like exploring the effect of a drug treatment for a disease in healthy (non-diseased individuals).

Is the work clearly and accurately presented and does it cite the current literature?

Partly

If applicable, is the statistical analysis and its interpretation appropriate?

Partly

Are all the source data underlying the results available to ensure full reproducibility?

Yes

Is the study design appropriate and is the work technically sound?

Yes

Are the conclusions drawn adequately supported by the results?

Partly

Are sufficient details of methods and analysis provided to allow replication by others?

Yes

Reviewer Expertise:

Weight loss, Obesity, Appetite regulation, body composition

I confirm that I have read this submission and believe that I have an appropriate level of expertise to confirm that it is of an acceptable scientific standard, however I have significant reservations, as outlined above.

# Response

**Published:** 2021-06-17

**Authors:** Michelle M Brown

**Affiliations:** Indiana University Bloomington, USA

We appreciate Dr. Astbury’s thoughtful comments. The comments reinforce that there are many details about recommendations to eat or skip breakfast that need to be considered beyond simplistic advice, including duration of intervention and to whom the advice is being supplied. Our prior revision addressed one such concern: whether eating versus skipping differs depending on baseline habits. Here, we supply an additional analysis accounting for duration of intervention and include more details about the participants in the original studies.

 Study (and, by extension, intervention) duration is important for the chronic management and prevention of obesity. Yet, all study duration thresholds for original studies and for meta-analysis inclusion are arbitrary. Our choice of 72 hours is arbitrarily short, but extends beyond just differences in eating (e.g., differences in weight from the actual digestive contents of eating versus skipping). Another meta-analysis arbitrarily chose 4 weeks as the cutoff, and we interpret Dr. Astbury’s remarks as suggesting 12+ weeks would be more reasonable. To address differences in study duration threshold for study inclusion, we now present a cumulative meta-analysis. This cumulative meta-analysis adds studies from the longest to the shortest duration. Studies (and strata within studies) are added by duration. Within a study duration (that is, if more than one study is of the same duration), studies and strata are added in an arbitrary order as organized in the data set. The cumulative meta-analysis including all studies of a particular duration or longer are represented by the last addition from top to bottom in the supplementary cumulative meta-analysis figure. Body weight and BMI had enough effect sizes to meaningfully synthesize results cumulatively across study durations. Using Dr. Astbury’s reasonable suggestion of 12 weeks, there is no significant differences between skipping and eating breakfast on body weight and BMI, as shown in the supplementary cumulative meta-analysis figure (see results in the row for Schlundt 1992s for body weight and Leidy 2015 for BMI). Using the 4 week cutoff used in another review, there is a significant estimate in favor of skipping, as shown in the supplementary cumulative meta-analysis figure (see the row for LeCheminant 2017). We emphasize that because this cumulative meta-analysis results in multiple testing, interpretation of any statistically significant results should be made with caution.

 Regarding the baseline obesity status, weight stability, and context of studies, we now include two new columns in Table 1. One column presents the baseline participant BMIs, and the other column presents information about weight stability, weight-related inclusion criteria, and context of the study. We discuss the importance of these considerations in the discussion section. Unfortunately, stratification by these factors is in some cases not easy to classify for a subgroup meta-analysis (e.g., baseline BMI categories are not harmonized across studies). Even in cases where classification seems possible, there are too few effect sizes to meaningfully synthesize.

 Given that our original analyses (including leave one out analyses), the stratified analyses by baseline breakfast habit submitted on the previous revision, and the cumulative analysis we submit with this revision were all generally robust to analytical decisions, we do not (and in many cases cannot) present additional quantitative syntheses on these important factors.

# Peer review: Reviewer response for version 1

**DOI:** 10.5256/f1000research.24742.r62393

**Published:** 2020-05-29

**Authors:** Enhad A. Chowdhury

**Affiliations:** 1 Department for Health, University of Bath, Bath, UK

**Overview**

 The work of Bohan Brown *et al *examines the impact of breakfast skipping versus consumption on obesity related anthropometric measures using meta-analytical methods. The authors have selected a focused question and I found the description of the methods and decisions behind inclusion/exclusion of papers to be thorough. As a non-statistician, I do not feel best placed to provide a critique of the statistical approach, but at face value do not see anything unusual. Generally, the data presentation was clear, and the interpretation of the results was appropriate to the scope of the work. I have outlined some specific comments for the authors consideration below, relating to small clarifications and the inclusion of a very recently published piece of work as point of context.

**Introduction**

**I found the introduction appropriate to set the context for the work.**

 As an area of nutrition that has been historically dominated by cross-sectional studies, it is of clear interest to examine the causal data relating to breakfast and body composition.

 I am not completely clear on the timings of the relative publications, but at time of review, the work by Bonnet referenced in the Prospero registration has now been published in Obesity. https://onlinelibrary.wiley.com/doi/abs/10.1002/oby.22791 ^1^ If possible, I would suggest this reference is added.

**Methods**

**I generally found the description of the methods to be thorough and clear.**

 “One breakfast skipping condition” and one “breakfast eating” condition. While the authors state regardless of modality – can I clarify whether this is based upon the original authors original definitions of these conditions (I assume this to be the case – but think it is useful to state here if so, in the same way that you have below in this section for weight status)?

 I found the description of included and excluded studies to be very thorough.

 As a non-statistician I do not consider myself well placed to examine the meta-analytical methods used thoroughly, this would be more appropriate for a statistician to evaluate. At face value, there does not seem to be anything particularly unusual about the approach employed.

**Results**

**I found the description of the results to be clear and thorough and the figures to be generally clear.**

 Figure 2 – I think it is helpful to have a visual representation of the different implementations of the interventions. However, is it possible to make this figure colour and make the different elements more distinct? With these very similar grey shades and fill patterns this figure is a little difficult to interpret.

 Figure 3 – I think it would be helpful to write in simple terms what each side of the 0 line represents on this figure within the figure legend, as you do for the description of Table 2. I also appreciate this may be for practical reasons, but the figures with only one missing value on the axis look a little strange – I presume this might be because the labels do not fit, but if easily adjustable I would suggest it might be beneficial to add this single missing label.

**Discussion**

**I generally found the discussion clear and highlighted appropriately the limitations based on the scope of the work.**

 As outlined for the introduction – the work of Bonnet is now published, and is potentially of interest, as they suggest based on their analysis a small but significantly reduced body weight (-0.54kg (CI: -1.05 to -0.03), which is also broadly in line with Sievert, suggesting a very small reduction in body mass with breakfast skipping. If possible, I would suggest this study is cited.

 I understand that clearly any effects in favour of breakfast skipping for body mass are small, but I think as the two other meta-analyses conducted conclude this, this is worth incorporating into your second paragraph about the potential for non-zero effects as a point of context. I don’t think this impacts upon your general point that any potential for effects are likely of low clinical significance.

 I think that with such a small set of studies with a very large variation between them, narrative reviews can often be valuable in this context for examining and considering the nuance of the results from different designs. I think it is good that you have clearly highlighted the limits of the meta-analytical approach used.

 I appreciate your point that longer term studies are often to determine the longer-term trajectory of interventions that may show short term impacts on body composition. Given that two other meta-analyses conclude that there may be a significant reduction overall (albeit small) with breakfast skipping is there also added value in determining if breakfast skipping over long durations causes more substantial weight loss?

 I am unclear as to the relevance of the “[j]ust because”? If this is a typo – it should be amended.

 While I think it is helpful that you have outlined the limitations of your analysis for informing the overall benefit of breakfast, I have two suggestions:

- I think it is relevant to acknowledge that breakfast consumption/omission has previously been shown to impact upon specific *components* of energy balance that may have independent effects upon health.
- I would suggest providing some references for studies/reviews examining the different factors that you highlight as not being examined in the present work (i.e. blood glucose control, cardiometabolic risk etc), should the reader be interested in those aspects.  

Is the work clearly and accurately presented and does it cite the current literature?

Yes

If applicable, is the statistical analysis and its interpretation appropriate?

I cannot comment. A qualified statistician is required.

Are all the source data underlying the results available to ensure full reproducibility?

Yes

Is the study design appropriate and is the work technically sound?

Yes

Are the conclusions drawn adequately supported by the results?

Yes

Are sufficient details of methods and analysis provided to allow replication by others?

Yes

Reviewer Expertise:

Energy balance, breakfast consumption, appetite regulation

I confirm that I have read this submission and believe that I have an appropriate level of expertise to confirm that it is of an acceptable scientific standard.

## References

[1] : Breakfast Skipping, Body Composition, and Cardiometabolic Risk: A Systematic Review and Meta-Analysis of Randomized Trials. *Obesity (Silver Spring)* .2020;28(6) : 10.1002/oby.22791 1098-1109 10.1002/oby.22791 32304359PMC7304383

# Response

**Published:** 2020-09-28

**Authors:** Michelle M Brown

**Affiliations:** Indiana University Bloomington, USA

***We thank Dr. Chowdhury for the feedback. We address the critiques below:***

***Regarding Bonnet et al, we have now added the reference. It was published between our first submission and our response to reviewers.***

***Regarding ‘one breakfast skipping condition’, we depend on the original authors’ definition where possible. We have expanded our exclusion criteria to give examples of situations where we would have excluded a breakfast/skipping group.***

***We have updated Figure 2. We prefer to avoid color to make figures color-blind friendly, and amenable to gray scale printing. We therefore have increased the contrast among three of the bars (light gray, dark gray, black), and increased the contrast and pattern of the one that previously had a dotted fill pattern. We hope this makes the figure more distinct.***

***We have updated the legend to Figure 3 to describe what left and right of zero means. We reprogrammed the code to generate the figures to make the tick marks consistent, rather than depending on the software defaults.***

***We now discuss Bonnet and Sievert’s meta-analyses in our discussion. It would seem that the instability of findings, including across meta-analyses that included only studies of longer duration, may warrant longer studies to confirm the effects of eating versus skipping on weight.***

***We changed the “[j]ust”, which was a stylistic artifact that was no longer needed.***

***We now cite papers that talk about outcomes beyond obesity, and we try to better emphasize that our analysis is limited to the binary eat versus skip breakfast on anthropometric measurements.***

# Peer review: Reviewer response for version 1

**DOI:** 10.5256/f1000research.24742.r62392

**Published:** 2020-05-27

**Authors:** Nerys Astbury

**Affiliations:** 1 Nuffield Department of Primary Care Health Sciences, NIHR Oxford Biomedical Research Centre, University of Oxford, Oxford, UK

This is a systematic review of randomised controlled trials comparing the consumption of breakfast with skipping breakfast and follows other similar SR on breakfast eating/skipping.

 There has been a long held debate regarding the potential effect of breakfast on energy intake, body weight and anthropometric measures, as the authors note this was driven by observational studies, but in recent years more RCT have emerged investigating effects.

 The methods are meretriciously reported and follow standard or gold standard procedures. Justification for some inclusion criteria should be included - to allow the reader to make a judgement whether the methods of this paper match its intended objectives. Since outcome is bodyweight/composition what is the purpose of including studies of 72hrs duration where likelihood of effect is minimal? 

 I feel that the discussion is weak and could be strengthened considerably. Limitations of this manuscript are not limited to the statistical methods and assumptions for dealing with missing data.

 Limitations of the current review should be discussed and critically analysed. Many of the studies included in this and other SR are small, and are often conducted in participants with a mixed usual breakfast habit who are not overweight or attempting to limit their energy intake or reduce their body weight. Further discussion on the clinical/research implications of this SR (and similar ones recently published) is warranted.

 The absence of is a substantial clinical trial of breakfast eating/skipping within the context of weight loss attempt is notable, could this be related to issues surrounding potential conflicts of interest and industry involvement?, -would be worth discussion. Some mention of contextual setting is important- it would be difficult to determine if eating/skipping breakfast aids/impairs weight loss if the participants in are not attempting to limit daily energy intake or weight loss. However breakfast may affect appetite regulation, thus aiding/impairing adherence to energy restricted diets (or it may not)? Could usual skippers made to eat have different response than usual consumers made to skip? Could sub-group differences be affecting ability to detect effects of breakfast on body weight and composition?

Is the work clearly and accurately presented and does it cite the current literature?

Partly

If applicable, is the statistical analysis and its interpretation appropriate?

Partly

Are all the source data underlying the results available to ensure full reproducibility?

Yes

Is the study design appropriate and is the work technically sound?

Yes

Are the conclusions drawn adequately supported by the results?

Partly

Are sufficient details of methods and analysis provided to allow replication by others?

Yes

Reviewer Expertise:

Weight loss, Obesity, Appetite regulation, body composition

I confirm that I have read this submission and believe that I have an appropriate level of expertise to confirm that it is of an acceptable scientific standard, however I have significant reservations, as outlined above.

# Response

**Published:** 2020-09-28

**Authors:** Michelle M Brown

**Affiliations:** Indiana University Bloomington, USA

***We thank Dr. Astbury for the feedback. We address the critiques below:***

***We have added to our discussion to outline more completely some of the limitations and choices we have made. We specifically address our choice of 72 hours, choices for defining breakfast and skipping, the fact that these effect estimates are pooled across weight loss backgrounds, weight categories, and breakfast compositions/timings. We have gone further to finish the subgroup analysis stratified by baseline breakfast habit. We initially presented a draft of the stratified analysis in our meeting abstracts. Completing it here addresses some of the reviewer’s concerns and allows us to uphold transparency by completely reporting analyses we have tried. This includes 5 new interaction-effect forest plots, a summary table, supplemental methods and results, and updated code.***

***We now discuss Bonnet and Sievert’s meta-analyses in our discussion, and briefly discuss some methodological and analytical differences that may explain differences in conclusions. We also highlight that these minor differences in analyses result in qualitatively different conclusions (i.e., differences in statistical significance), which reinforces the overall uncertainty in conclusions that can be drawn from this body of evidence.***

***We hope that these additions and clarifications, in addition to the limitations we initially discussed about length of studies, baseline breakfast habits, types of breakfast, and other considerations, more fully orient the reader to the limitations both of our analysis and the evidence base for these questions.***
